# The Impact of Tick-Borne Diseases on the Bone

**DOI:** 10.3390/microorganisms9030663

**Published:** 2021-03-23

**Authors:** Imran Farooq, Tara J. Moriarty

**Affiliations:** 1 Faculty of Dentistry, University of Toronto, Toronto, ON M5G 1G6, Canada; imran.farooq@mail.utoronto.ca; 2 Department of Laboratory Medicine and Pathobiology, Faculty of Medicine, University of Toronto, Toronto, ON M5G 1G6, Canada

**Keywords:** tick, infection, bone, virus, *Anaplasma*, *Ehrlichia*, *Babesia*, *Borrelia*

## Abstract

Tick-borne infectious diseases can affect many tissues and organs including bone, one of the most multifunctional structures in the human body. There is a scarcity of data regarding the impact of tick-borne pathogens on bone. The aim of this review was to survey existing research literature on this topic. The search was performed using PubMed and Google Scholar search engines. From our search, we were able to find evidence of eight tick-borne diseases (Anaplasmosis, Ehrlichiosis, Babesiosis, Lyme disease, Bourbon virus disease, Colorado tick fever disease, Tick-borne encephalitis, and Crimean–Congo hemorrhagic fever) affecting the bone. Pathological bone effects most commonly associated with tick-borne infections were disruption of bone marrow function and bone loss. Most research to date on the effects of tick-borne pathogen infections on bone has been quite preliminary. Further investigation of this topic is warranted.

## 1. Introduction

A wide range of bacterial, viral, and protozoan pathogens can be transmitted by ticks, which act as vectors transporting pathogens to hosts, including humans [1,2]. These pathogens are responsible for many known human diseases, including those described in Table 1 below.

**Figure 1:**
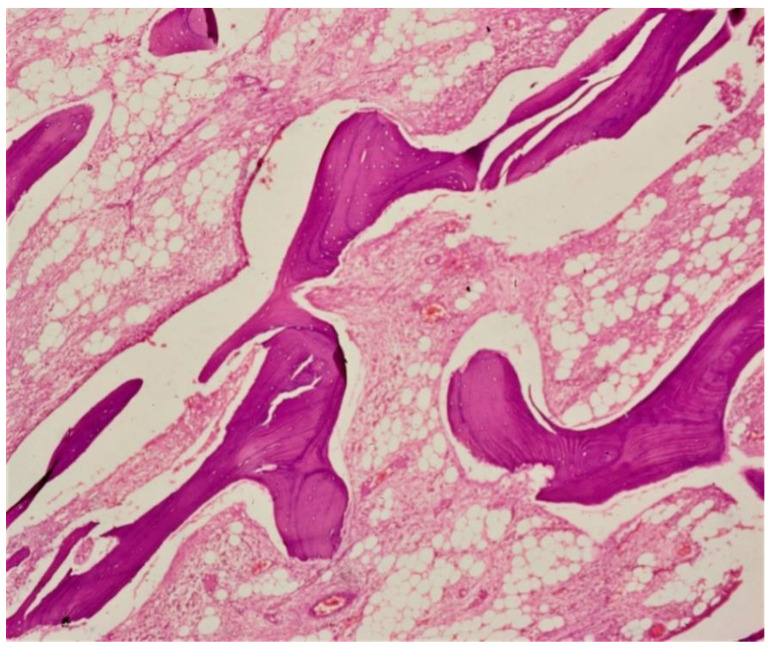
Hematoxylin and eosin (H and E) stained decalcified section showing bony trabeculae of spongy bone with marrow spaces and fat tissue.

One of the most essential structures in vertebrates is bone, which supports the body, protects vital organs and stores minerals [23]. Bone is a mixture of inorganic content (minerals primarily in the form of hydroxyapatite crystals), organic components such as collagen, cells and proteins, and water [24,25]. There are two major types of bone: (1) cortical (compact) and (2) trabecular (spongy, cancellous or porous). In trabecular bone, the spaces between mineralized bone trabecula are filled with bone marrow and fat tissue (Figure 1). Bone marrow is highly vascularized and innervated and is responsible for the production of red blood cells, granulocytes, platelets, monocytes and lymphocytes [26].

**Figure 2:**
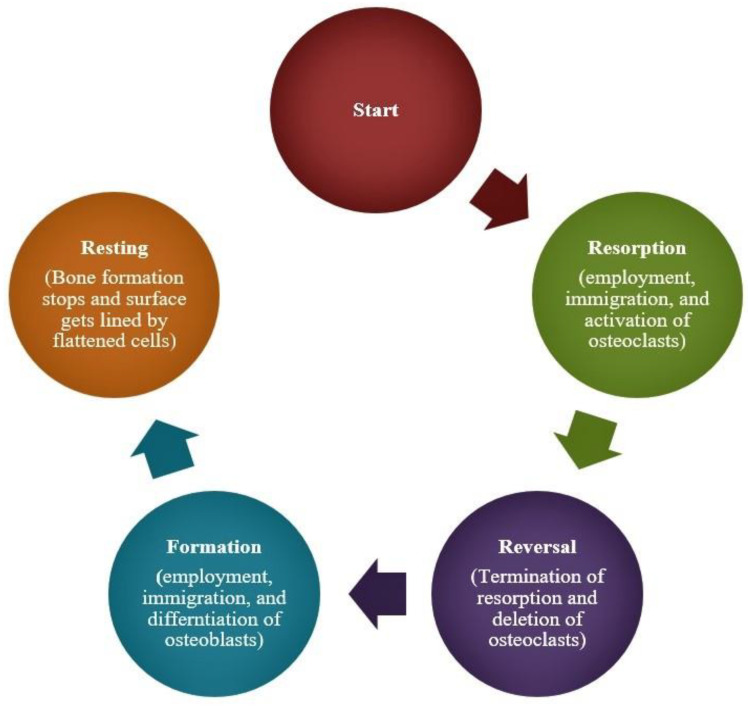
Flow chart depicting sequence of events during bony remodelling process. Adapted from Raggat and Partridge [29].

Bone in most parts of the body is renewed by a dynamic process called bony remodelling [27], which involves four basic steps: resorption, reversal, formation and resting (Figure 2). Bony remodelling is driven by counterbalancing activities of osteoclasts, which are responsible for bone resorption, and osteoblasts, which are responsible for bone building (apposition) [28].

Bone plays a complex and important role in immune responses, and local and systemic infections can also cause bone pathology [30,31,32]. Osteoblasts and osteocytes can regulate numbers and differentiation of B-cells and T-cells in bone marrow [33,34], and osteoclasts create the bone marrow cavity required for normal hematopoiesis [35]. Infections and conditions accompanied by systemic immune responses, such as inflammatory bowel disease, can cause bone loss because inflammatory cytokines can stimulate osteoclastogenesis and bone resorption [36]. Microbes also directly colonize bone, as a result of injury, surgery, implanted devices and hematogenous dissemination from more distant infection sites. In some cases, colonization is accompanied by infectious pathologies including bone marrow dysfunction and bone loss.

To the best of our knowledge, the effects of tick-borne infections on bone have not been reviewed previously. We surveyed the primary research literature for studies investigating the changes occurring in the bone structure and function during human tick-borne pathogen infections. We used PubMed and Google Scholar search engines and keywords “tick”, “vector”, “bone” and the names of individual human tick-borne diseases and pathogens. Conference abstracts and articles published in languages other than English were excluded. These searches retrieved 500+ unique results, of which 132 were finally selected based on their relevance to the investigated topic in this review. Tick-borne diseases associated with human bone phenotypes are listed in Table 2 and described in greater detail below.

## 2. Anaplasmosis (Formerly Human Granulocytic Ehrlichiosis)

Anaplasmosis [3] is caused by the Gram-negative intracellular bacterium *A. phagocytophilum*, which infects myeloid cells (neutrophils, megakaryocytes, mast cells) and endothelial cells [37,38,39]. *A. phagocytophilum* and related species can infect humans, cattle, deer, dogs, foxes, horses, wild and laboratory mice and sheep [40,41,42]. In humans, the most common signs and symptoms include fever, malaise, myalgia, headache, arthralgia, thrombocytopenia, leukopenia and less commonly anemia [3]. Complications can include respiratory illness, organ failure and death. Although *A. phagocytophilum* appears to typically be transmitted by ticks, transmission has been reported after contact with infected blood [43].

*A. phagocytophilum* is detected in the bone marrow of sheep, mice, deer, horses, dogs, and humans [40,41,42,44,45,46,47,48]. Infection in mice and humans often features peripheral cytopenias accompanied by bone marrow abnormalities associated with dyserythropoiesis, dysmegakaryopoiesis impaired red blood cell and platelet regeneration) and hemophagocytic lymphohistiocytosis [41,47,48,49,50,51,52,53]. There is some evidence that these cytopenias could result from peripheral processes including extravascular hemolysis [54], but peripheral cytopenias appear to primarily result from dyserythropoiesis and dysmegakaryopoiesis [41,47,48]. The molecular mechanisms underlying bone marrow dysfunction in anaplasmosis are not yet understood. In vitro, bone marrow progenitors belonging to monocytic and granulocytic lineages are prone to infection by *A. phagocytophilum* [55]. Animal studies suggest that myelosuppressive chemokines produced during anaplasmosis reduce bone marrow proliferation and differentiation [56]. The exposure of bone marrow cells to chemokines (IL-8 and MIP-1) decreases the proliferation and differentiation of myeloid progenitor cells leading to reduced hematopoiesis [57]. Substantial further research is needed to understand the mechanisms of bone marrow suppression and their consequences in disease progression and outcomes of anaplasmosis. 

## 3. Ehrlichiosis

Ehrlichiosis is caused by multiple species from the *Ehrlichia* genus of obligate intracellular bacteria, including *E. chaffeensis*, *E. ewingii* and *E. muris eauclairensis* [58,59]. *Ehrlichia* mainly infects monocytes and neutrophils in dogs, rodents, and humans [8,60]. The signs and symptoms of ehrlichiosis are non-specific, similar to many tick-borne infections and include fever, chills, and a rash [61].

*Ehrlichia* have been detected in the bone marrow of dogs, cows, mice, and humans [62,63,64,65]. Infections in animals and humans feature monocytosis and cytopenias accompanied by abnormal bone marrow function, indicated by dyshematopoiesis and dyserythropoiesis [66,67,68]. Peripheral cytopenias marked by anemia, thrombocytopenia or neutropenia have been reported in animals and humans [62,63,64,65,66,67,68]. Although the mechanism by which ehrlichiosis causes cytopenias is not well established, it is believed that *Ehrlichia* can invade myeloid cells of the bone marrow, resulting in dyshematopoiesis causing cytopenias [69]. As a compensatory response to the developing cytopenias, an increased number of immature megakaryocytes are produced [70], possibly causing thrombocytopenia. Cyotopenias observed in ehrlichiosis could also result from hypocellular bone marrow [70], although the reason for its hypocellularity is not clear. During chronic *Ehrlichia* infections, cytokine-mediated immune suppression, decreased production of blood cells, and sequestration of erythrocytes can all play their part in decreased erythropoiesis [68]. Bone marrow abnormalities seen in ehrlichiosis could also be caused by the production of type I interferons (IFNα/β) that are produced in response to almost all the infections. During ehrlichial infections, IFNα/β induces bone marrow loss and impaired hematopoiesis by causing decreased proliferation of hematopoietic stem and progenitor cells [69]. Though an effort has been made to pinpoint the cause of bone marrow suppression during ehrlichiosis, these mechanisms are largely speculative, and further work is needed to determine their exact effects on the bone marrow causing dyshematopoiesis. 

## 4. Babesiosis

Microscopic intracellular *Babesia* parasites cause babesiosis. *Babesia* species mostly infect erythrocytes in the host, causing haemolytic anemia, which is especially dangerous in older adults [71]. Mild to moderate forms of illness are usually accompanied by fever, fatigue, malaise, headache, and chills [72]. Complications of severe illness include respiratory distress, renal failure, coma, or death [72]. 

*Babesia* have been detected in bone marrow of cattle, mice, dogs, and humans [73,74,75,76,77]. *Babesia* are intraerythrocytic parasites [78] and the most significant bone marrow abnormality associated with babesiosis is dyserythropoiesis, leading to anemia [79]. Thrombocytopenia also occurs in both animals and humans but is a less common presentation than anemia in both species [80,81,82,83]. *Babesia* invasion of erythrocytes can lead to intravascular hemolysis [80]. The mechanisms by which *Babesia* suppresses bone marrow function are largely unknown.

## 5. Lyme Disease

Members of the *B. burgdorferi* species complex are extracellular spirochete bacteria that cause Lyme disease or Lyme borreliosis [11]. Early Lyme disease symptoms can include fever, chills, headache, sweating, joint pain, myalgia, swollen lymph nodes and erythema migrans skin rash. Untreated Lyme disease can have complications such as arthritis, endocarditis, and neuroborreliosis [11]. 

*B. burgdorferi* has been detected in the bone marrow of dogs, birds, mice and humans [84,85,86,87], and bone pain, erosion at articular surfaces, osteomyelitis and osteopenia have been reported [86,87,88,89,90,91,92,93,94,95,96,97]. *B. burgdorferi* infection in mice causes trabecular bone loss due to inhibition of bone apposition rather than bone resorption [86]. One plausible reason for this finding could be that *B. burgdorferi* infection causes an upregulation of tumor necrosis factor-alpha (TNF-α), IL-1, and IL-6 [98,99] that can cause suppression of osteoblastogenesis [100]. The bone infection caused by *B. burgdorferi* is an emerging area and requires further investigation to shed light on the exact mechanism behind this phenomenon, especially in terms of its effect on osteoblastogenesis. 

## 6. Bourbon Virus Disease

Bourbon virus disease is caused by the recently discovered Bourbon virus [101]. Signs and symptoms of this disease resemble those of many other tick-borne diseases and include fever, sweating, headache, fatigue, myalgia and arthralgia [101]. 

Bourbon virus infection in humans and mice often features peripheral cytopenias including thrombocytopenia, leukopenia and lymphopenia indicating possible bone marrow suppression [101,102]. As Bourbon virus disease has been recently discovered, more investigation is needed to understand its impact on the bone.

## 7. Colorado Tick Fever Disease

Colorado tick fever virus (CTFV) can infect humans, rodents and some other mammals and is responsible for the rare CTF disease in humans [103,104,105]. CTF disease signs and symptoms include biphasic fever, chills, headache, fatigue, skin rash and peripheral cytopenias including leukopenia, anemia and thrombocytopenia [106,107].

CTFV can infect and persist in human and murine erythrocytes for extended periods [108]. The prolonged viremia associated with CTFV infection is possibly due to the prolonged persistence of CTFV in intra-erythrocytic locations [109]. CTFV can cause multilineage cytopenias by directly invading and replicating inside the bone marrow CD34+ stem cells [110]. As CD34+ stem cells are important part of the hematopoietic system [111], the replication of CTFV inside these cells is indicative of abnormal hematopoiesis and bone marrow suppression [110]. The CTFV infection can also affect the immature bone marrow cells and this infection can persist through their various stages of maturation [112]. Although various reasons for bone marrow suppression have been hypothesized in the literature, more investigative studies are recommended.

## 8. Tick-Borne Encephalitis

The tick-borne encephalitis (TBE) is caused by the TBE virus (TBEV) [113]. Many patients infected by TBEV remain asymptomatic. Symptomatic patients usually suffer from fever, headaches, body aches, malaise, nausea, and vomiting, with thrombocytopenia and leukopenia seen early in infection in humans, dogs and horses [114,115,116,117,118,119,120].

TBE virus has been detected in the bone marrow of animals and humans [121,122], but it is unknown if and how bone marrow infection contributes to peripheral cytopenias.

## 9. Crimean–Congo Hemorrhagic Fever 

Crimean–Congo Hemorrhagic Fever (CCHF) is caused by the CCHF virus (CCHFV) [123]. Infection can result in multi-organ failure secondary to cytokine storm and hemorrhage and has a fatality rate ranging between 3% and 50% [124]. The most common signs and symptoms of CCHF include sudden fever, chills, and severe migraine-like headaches [125]. Less common symptoms are vomiting and haemorrhages [126].

CCHFV presence in the bone marrow is not reported in the literature. CCHFV infection features peripheral cytopenias that are marked by thrombocytopenia and leukopenia in animals and humans [127,128,129,130]. One fatal feature of CCHFV infection is hemophagocytic syndrome (HPS) that is characterized by excessive bleeding due to cytokine storm [131]. Uncontrolled hypercytokinemia leads to myelosuppression and vascular damage causing multiple organ failure and death [132]. It is not clear if peripheral cytopenias are secondary to bone marrow dysfunction or systemic immune pathologies, and more research is needed on this topic. 

## 10. Conclusions

This review concludes that multiple tick-borne diseases can infect and cause pathology in bone and bone marrow. Mechanisms underlying bone pathology in many of these diseases have been under-investigated and further study of this topic is warranted. 

## Figures and Tables

**Table 1:** Human diseases caused by tick-borne pathogens.

Bacterial	Viral	Parasitic
**Anaplasmosis** (*Anaplasma phagocytophilum*) [3]	**Nairoviral diseases:** Crimean-Congo Hemorrhagic Fever (*CCHFV*) [4]; Nairobi Sheep Disease (*NSDV*) [5]; Songling Virus Disease (*SGLV*) [6]	**Babesiosis** (*Babesia microti*, *B. divergens*, *B. duncani*, *B. venatorum*) [7]
**Ehrlichiosis** (*Ehrlichia chaffeensis*, *E. ewingii*, *E. muris*) [8]	**Phenuiviral diseases:** Heartland Virus Disease (*HRTV*) [9]; Severe Fever with Thrombocytopenia Syndrome (*SFTSV*) [10]	
**Lyme Disease** (*Borreliella afzelii*, *B. burgdorferi sensu stricto*, *B. garinii*, *B. mayonii*) [11]	**Orthomyxoviral diseases:** Bourbon virus disease (*BRBV*) [12]	
**Rickettsioses**, including Flinders Island (*R. honei*), Israeli (*R. conoriisubsp. israelensis*), Mediterranean (*R. conoriisubsp. conorii*), Japanese (*R. japonica*) and Rocky Mountain (*R. rickettsii*) Spotted Fevers; Indian (*R. conoriisubsp. indica*), Queensland (*R. australis*) and Siberian Tick Typhus (*R. sibiricasubsp. sibirica*); Far Eastern (*R. heilongjiangensis*) and Lymphangitis-Associated (*R. sibirica subsp. mongolitimonae*) Rickettsioses; African Tick Bite *(R. rickettsii)* and Astrakhan (*R. conorii* *subsp. caspia*) Fevers; SENLAT (*R. raoultii*) [13,14]	**Flaviviral diseases** [15]: Alkhurma Hemorrhagic Fever (*AHFV*) [16]; Kyasanur Forest Disease (*KFDV*) [17]; Omsk Hemorrhagic Fever (*OHFV*) [15]; Powassan Disease (*POWV*) [15,18]; Tick-borne Encephalitis (*TBEV*) [19]	
**Tick-borne Relapsing Fever** (*Borrelia crocidurae*, *B. duttoni*, *B. hermsii*, *B. hispanica*, *B. miyamotoi*, *B. parkeri*, *B. persica*, *B. turicatae*) [20]	**Reoviral diseases**: Colorado Tick Fever Disease (*CTFV*); Eyach Virus Disease (*EYAV*) [21]	
**Tularemia** (*Francisella tularensis*) [22]		

**Table 2:** Tick-borne diseases with reported human bone phenotypes.

Tick-Borne Disease	Impact on Bone	
	Disrupted Bone Marrow Function	Bone Loss
Anaplasmosis	√	-
Ehrlichiosis	√	-
Babesiosis	√	-
Lyme disease	-	√
Bourbon virus disease	√	-
Colorado tick fever disease	√	-
Tick-borne encephalitis	√	-
Crimean-Congo Hemorrhagic Fever	√	-
